# Complexities of a protonatable substrate in measurements of Hoechst 33342 transport by multidrug transporter LmrP

**DOI:** 10.1038/s41598-020-76943-0

**Published:** 2020-11-18

**Authors:** Brendan M. Swain, Dawei Guo, Himansha Singh, Philip B. Rawlins, Mark McAlister, Hendrik W. van Veen

**Affiliations:** 1 Department of Pharmacology, University of Cambridge, Tennis Court Road, Cambridge, CB2 1PD UK; 2 Structure, Biophysics and Fragment-Based Lead Generation, Discovery Sciences, BioPharmaceuticals R&D, AstraZeneca, Cambridge, CB4 0WG UK

**Keywords:** Antimicrobial resistance, Cellular microbiology

## Abstract

Multidrug transporters can confer drug resistance on cells by extruding structurally unrelated compounds from the cellular interior. In transport assays, Hoechst 33342 (referred to as Hoechst) is a commonly used substrate, the fluorescence of which changes in the transport process. With three basic nitrogen atoms that can be protonated, Hoechst can exist as cationic and neutral species that have different fluorescence emissions and different abilities to diffuse across cell envelopes and interact with lipids and intracellular nucleic acids. Due to this complexity, the mechanism of Hoechst transport by multidrug transporters is poorly characterised. We investigated Hoechst transport by the bacterial major facilitator superfamily multidrug-proton antiporter LmrP in *Lactococcus lactis* and developed a novel assay for the direct quantitation of cell-associated Hoechst. We observe that changes in Hoechst fluorescence in cells do not always correlate with changes in the amount of Hoechst. Our data indicate that chemical proton gradient-dependent efflux by LmrP in cells converts populations of highly fluorescent, membrane-intercalated Hoechst in the alkaline interior into populations of less fluorescent, cell surface-bound Hoechst in the acidic exterior. Our methods and findings are directly relevant for the transport of many amphiphilic antibiotics, antineoplastic agents and cytotoxic compounds that are differentially protonated within the physiological pH range.

## Introduction

Hoechst 33342 (referred to as Hoechst) is a bis-benzamide dye that is relatively non-toxic and cell-permeable, making it suitable for a wide range of applications in cell biology. As its fluorescence increases when bound to adenine–thymine-rich sequences in the minor groove of double-stranded DNA, Hoechst is commonly used to stain nuclei, track chromatin condensation, and monitor the cell cycle phase in eukaryotic cells^1–3^. Hoechst fluorescence also increases when the dye intercalates between lipid molecules in biological membranes^4^. Hoechst is a substrate of multidrug transporters, which translocate a wide range of structurally unrelated compounds from cells in a metabolic energy-dependent fashion^4–6^ and reduce Hoechst fluorescence in the transport process. In various mammalian cell lines and tissues, Hoechst efflux by the ATP-binding cassette (ABC) multidrug transporters ABCB1 and ABCG2 shows the presence of a ‘side population’ with decreased Hoechst fluorescence^5,7^. The interactions of bacterial multidrug transporters with Hoechst is also documented in a wide range of publications. For example, Hoechst was used in studies of drug efflux-based antibiotic resistance in *Salmonella enterica* serovar Typhimurium^8,9^ and *Acinetobacter baumannii*^10,11^, and in transport measurements for the ABC multidrug transporters LmrA from *Lactococcus. lactis*^12–14^, Sav1866 from *Staphylococcus aureus*^15^, MsbA and YbhFSR from *E. coli*^16,17^, and PatAB from *Streptococcus pneumoniae*^18^. Hoechst transport assays were used in studies of secondary-active multidrug and toxic compound extrusion (MATE) transporters VcmA from *Vibrio cholerae*^19^ and AbeM from *A. baumannii*^20^, and in studies of a novel natural product inhibitor of the major facilitator superfamily (MFS) multidrug transporter NorA from *Staphylococcus aureus*^21^.

Hoechst transport has also been reported for the MFS multidrug transporter LmrP from *L. lactis*, which can efflux a wide range of clinically relevant antibiotics and cytotoxic compounds and Ca^2+^ from cells^22,23^. LmrP exports monovalent cationic ethidium and divalent cationic propidium by electrogenic exchange with 2 protons and 3 protons, respectively^24,25^. These transport reactions are dependent on the transmembrane chemical proton gradient (ΔpH, interior alkaline) and membrane potential (Δψ, interior negative) components of the proton-motive force (PMF = Δψ − ZΔpH in which Z = 59 mV at 24 °C). Therefore, the coupling stoichiometry in LmrP is variable and dependent on the charge and physico-chemical properties of the substrate. This phenomenon reflects the different mechanisms of drug binding in the interior chamber of LmrP, some of which alter the availability of catalytic carboxylates for proton interactions^24,25^. Hoechst transport and binding have also been used to characterise drug interactions in LmrP^6,23,26^, to define steps in the transport cycle in structural analyses^27^ and, most recently, to trap the protein in a conformation that could be crystallised, thus allowing the determination of the three-dimensional protein structure^28^.

In spite of the frequent use of Hoechst as a reporter for drug resistance and efflux, the quantitative interpretation of the transport-associated fluorescence change of Hoechst is unclear and complicated by two significant factors. Firstly, complex equilibria will exist between pools of Hoechst in the cytoplasm, bound to DNA and intercalated in biological membranes, in which the dye exhibits different levels of fluorescence emission. Secondly, three basic nitrogen atoms are present in Hoechst’s chemical structure. The protonation state of Hoechst, therefore, varies within the physiological range of pH inside and outside of cells, leading to pH-dependent fluorescence and interaction with macromolecular structures^29^. Measurements of Hoechst transport are further complicated for LmrP and other proton-coupled multidrug transporters, where the pH difference across the plasma membrane provides a driving force for Hoechst extrusion by drug/proton antiport.

To facilitate the quantitative analysis of Hoechst transport by LmrP and deconvolute the physical and environmental effects on Hoechst fluorescence, we investigated the fluorescence properties of dissolved, and lipid or DNA-bound Hoechst as a function of pH, and developed an assay for the direct determination of the amount of Hoechst associated with cells that is independent of its fluorescence in situ. We compared this assay to the conventional cell-based fluorescence measurements of LmrP-mediated Hoechst transport in *L. lactis*.

## Results

### Hoechst fluorescence in DNA solutions

**Figure 1:**
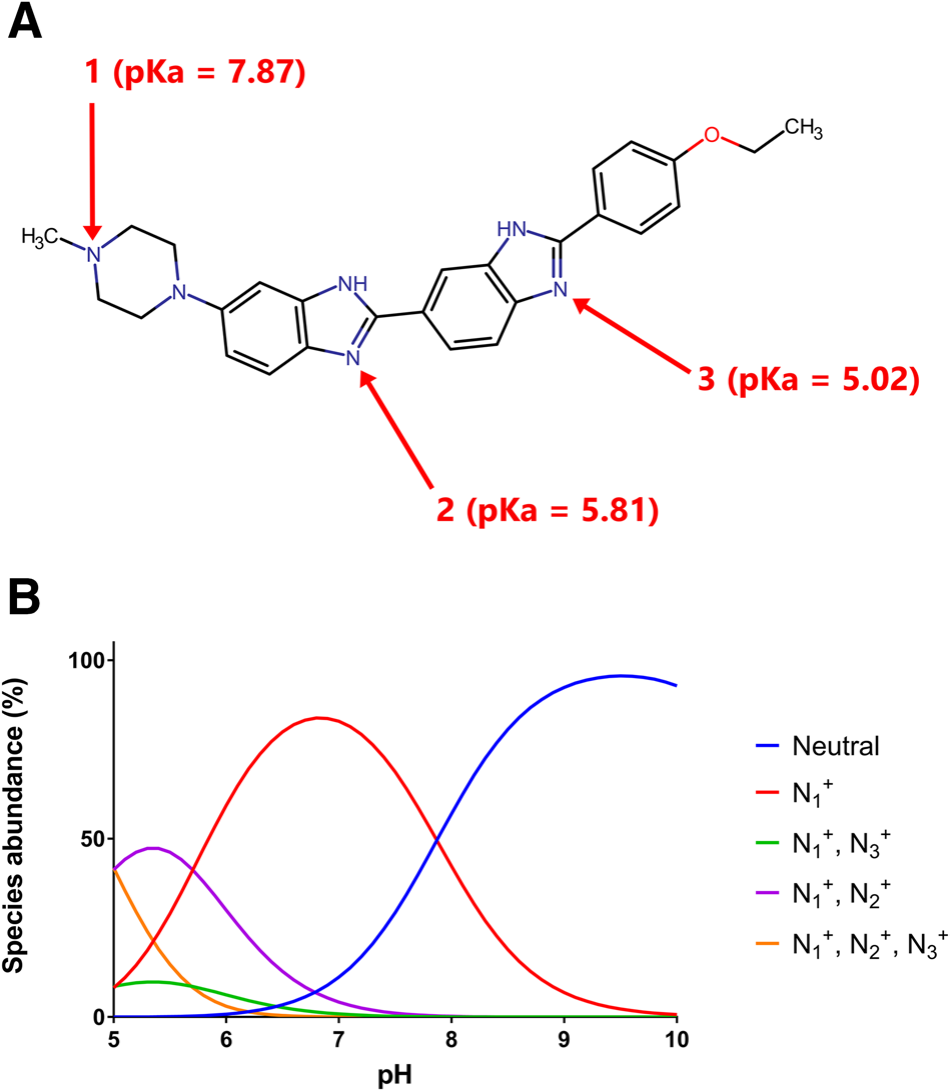
Protonation states of Hoechst. **(A)** Structure of Hoechst 33342 in which the three nitrogen atoms with predicted changes in protonation in the physiological pH range are labelled. **(B)** Calculated distribution of Hoechst species at varying pH. For simplicity in presentation, species with a maximum prevalence of less than 1% are not shown.

**Figure 2:**
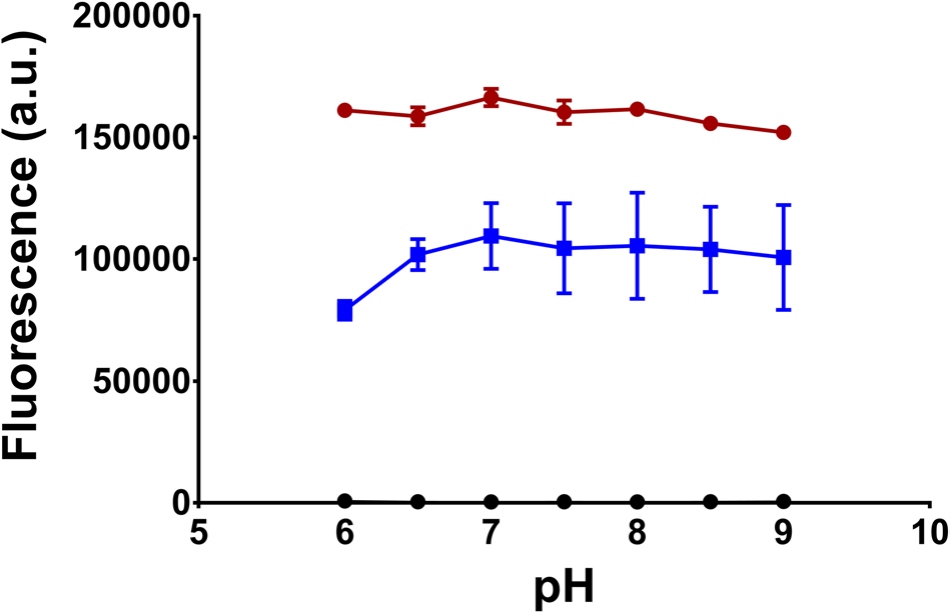
Hoechst and ethidium fluorescence in buffer at different pH values. Fluorescence of 1 µM Hoechst without DNA (black circles) or with 1 mg mL^−1^ sheared calf thymus DNA (blue squares), or fluorescence of 2 µM ethidium bromide in the presence of the DNA (red circles) was measured in 20 mM (K)MES-PIPES-HEPES buffer set at the pH values indicated in the figure. Error bars represent mean ± S.E.M of n = 3 independent experiments. The error bars for some of the data points were too small to be displayed and are hidden behind the data point symbols.

Hoechst 33342 contains three basic nitrogen atoms (Fig. 1A), the predicted pKa values of which (pKa N_1_ 7.87, N_2_ 5.81 and N_3_ 5.02) fall well within the pH range supporting the growth of *L. lactis*^30^. The predicted species distribution at varying pH values (Fig. 1B) suggests that protonated cationic species of Hoechst dominate at low and neutral pH, with the proportion of the neutral form increasing with alkalinity. These calculations agree well with the published speciation of Hoechst 33342 and Hoechst 33258^29,31,32^. As Hoechst can interact with various macromolecular targets in the cell, we first measured the baseline fluorescence of Hoechst in 20 mM (K)MES-PIPES-HEPES buffer with fixed pH values in a range from 6.0 to 9.0 (Fig. 2). However, the relative fluorescence intensity of the baseline was negligible when compared to that in the presence of 1 mg mL^−1^ sheared calf thymus DNA, the minor groove of which is a target for Hoechst binding (Fig. 2). The fluorescence of Hoechst in the DNA solution increased with increasing buffer pH and reached a maximum at around pH 7.0, above which fluorescence remained relatively constant. Of note was the increased error in the measurements of Hoechst fluorescence at alkaline pH, which might relate to the decreased solubility of the dye. The fluorescence of monovalent cationic ethidium, which does not accept or release protons in the physiological pH range and which binds to DNA by intercalation, was used for comparison (Fig. 2). There was little variation in the fluorescence of DNA-bound ethidium with buffer pH, supporting the notion that pH-induced changes in fluorescence are Hoechst-specific and not caused by general pH-induced changes in the structure of DNA. The pH profile of the fluorescence emission of Hoechst bound to DNA is remarkably similar to the abundance of Hoechst species that remain unprotonated at N_2_ or N_3_ (Figs. 1 and 2). This is consistent with results from X-ray crystallography, where the equivalent atoms in Hoechst 33258 were shown to be involved in hydrogen bonding within the minor groove of DNA^33^. This interaction is disrupted by protonation at N_2_ or N_3_, leading to the observed negative correlation between fluorescence and prevalence of these Hoechst species (Fig. 2). As the fluorescence emission of free Hoechst in solution is very low compared to that of DNA-bound Hoechst (Fig. 2), the contribution of free Hoechst to total Hoechst fluorescence in cells is negligible.

### Hoechst fluorescence in liposome suspensions

**Figure 3:**
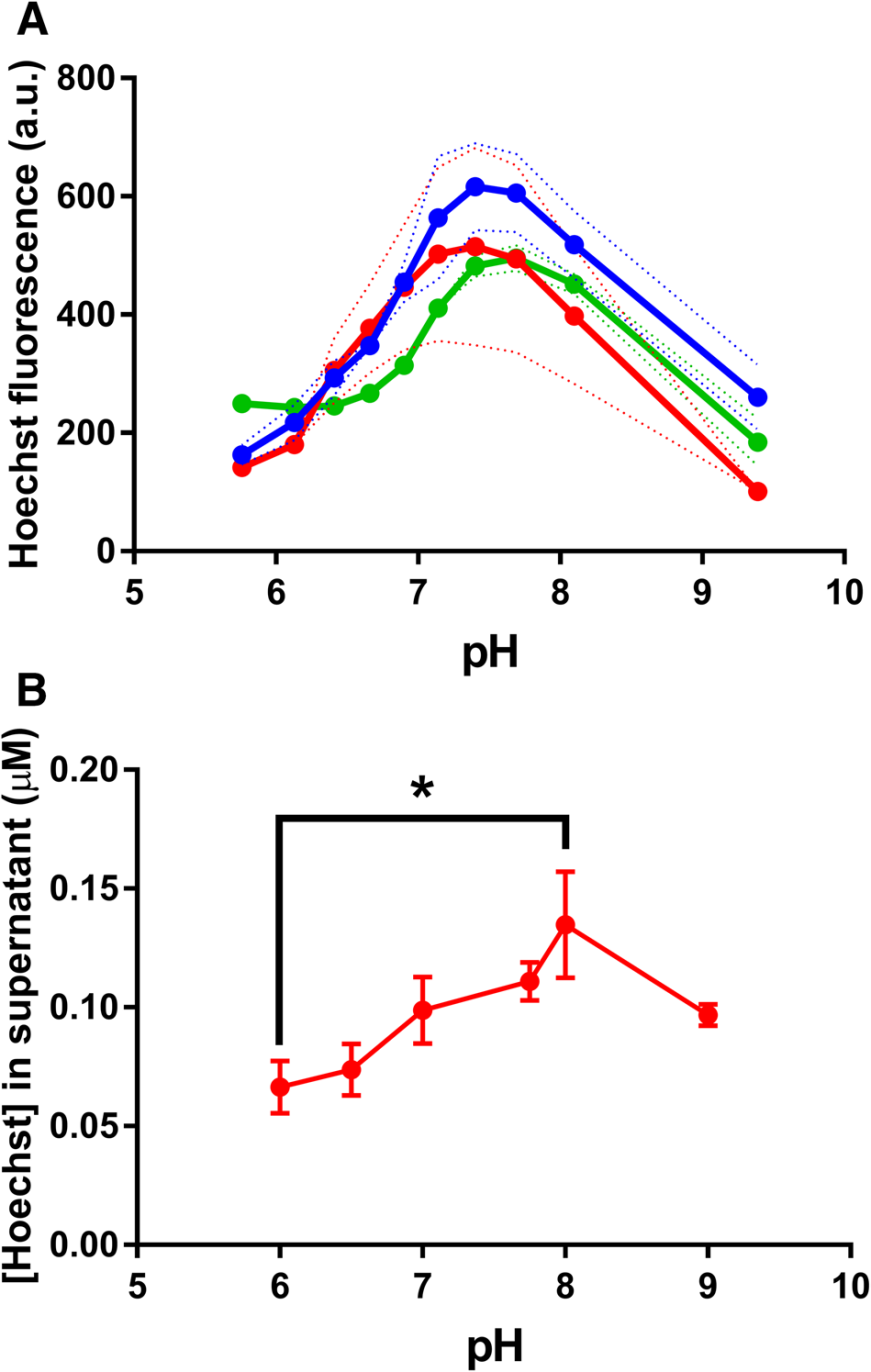
The fluorescence of Hoechst in the presence of (proteo)liposomes. **(A)** Liposomes without (blue circles) or with reconstituted LmrP (red circles), or liposomes containing 1 mg mL^−1^ DNA in the lumen (green circles), were diluted 100-fold in Assay Buffer (20 mM (K)MES-PIPES-HEPES pH 5.8) containing 1 µM nigericin. Following the addition of 0.1 µM Hoechst, the dye fluorescence was measured. Subsequently, the buffer pH was increased stepwise to fixed values from 5.8 to 9.5 by additions of 5 M KOH. The Hoechst fluorescence was re-measured at each step. **(B)** Empty liposomes were diluted 20-fold in Assay Buffer containing 1 µM nigericin and 0.1 µM Hoechst at fixed pH values as indicated in the figure. After ultracentrifugation, the supernatant was collected and mixed 1:1 with isopropanol to measure Hoechst fluorescence. The absolute Hoechst concentration in the supernatant was calculated by comparison with samples containing known concentrations of Hoechst in Assay Buffer at fixed pH values between 6.0 and 9.0. Solid and dotted lines in panel A and data in panel B represent mean ± S.E.M of n = 3 independent experiments (one-way analysis of variance; * p < 0.05).

We also measured the fluorescence of Hoechst in liposomes containing *E. coli* lipids and phosphatidylcholine. At fixed pH_OUT_ values, where pH_OUT_ = pH_IN_, the Hoechst fluorescence increased with increasing pH to a maximum at approximately pH 7.5 (Fig. 3A). After removal of the liposomes by centrifugation, there was a significantly larger quantity of Hoechst remaining in the supernatant at pH 8.0 than at pH 6.0 (Fig. 3B). Thus, while dye binding to the membrane reduces with increasing pH, the fluorescence output per molecule of liposome-associated Hoechst increases dramatically with increasing pH. Given the species distribution of Hoechst as a function of pH (Fig. 1B) and the high fluorescence emission of Hoechst in a hydrophobic environment, these data suggest that, at alkaline pH, the neutral form and monovalent cation form with protonation at the N_1_ position (in the tetrahydropyrazine moiety) can intercalate between the hydrophobic phospholipids in the membrane. At acidic pH, the divalent cationic form with protonation at N_1_ and N_2_ (in the first benzimidazole moiety) and trivalent form with protonation at N_1_, N_2_ and N_3_ (in the second benzimidazole moiety) intercalate less deeply in the membrane, or not at all. These forms adsorb onto the membrane surface where they remain exposed to the aqueous solvent, resulting in low fluorescence emission.

Neither the reconstitution of the MFS multidrug transporter LmrP into the liposomes nor the inclusion of 1 mg mL^−1^ of DNA in the liposomal lumen had statistically significant effects on the Hoechst fluorescence at any pH (Fig. 3A). Due to the high lipid-to-DNA ratio of 500:1 (w/w) in this membrane system, the fluorescence of membrane-intercalated Hoechst is dominant over that of DNA-bound Hoechst. With a lipid-to-DNA ratio of approx. 7:3 (w/w) in gram-positive bacterial cells^34^, the intercalation of Hoechst in the membrane is the major contributor to the total Hoechst fluorescence in this cellular environment, although Hoechst binding to DNA should also be considered.

### Hoechst fluorescence in bacterial cell suspensions

**Figure 4:**
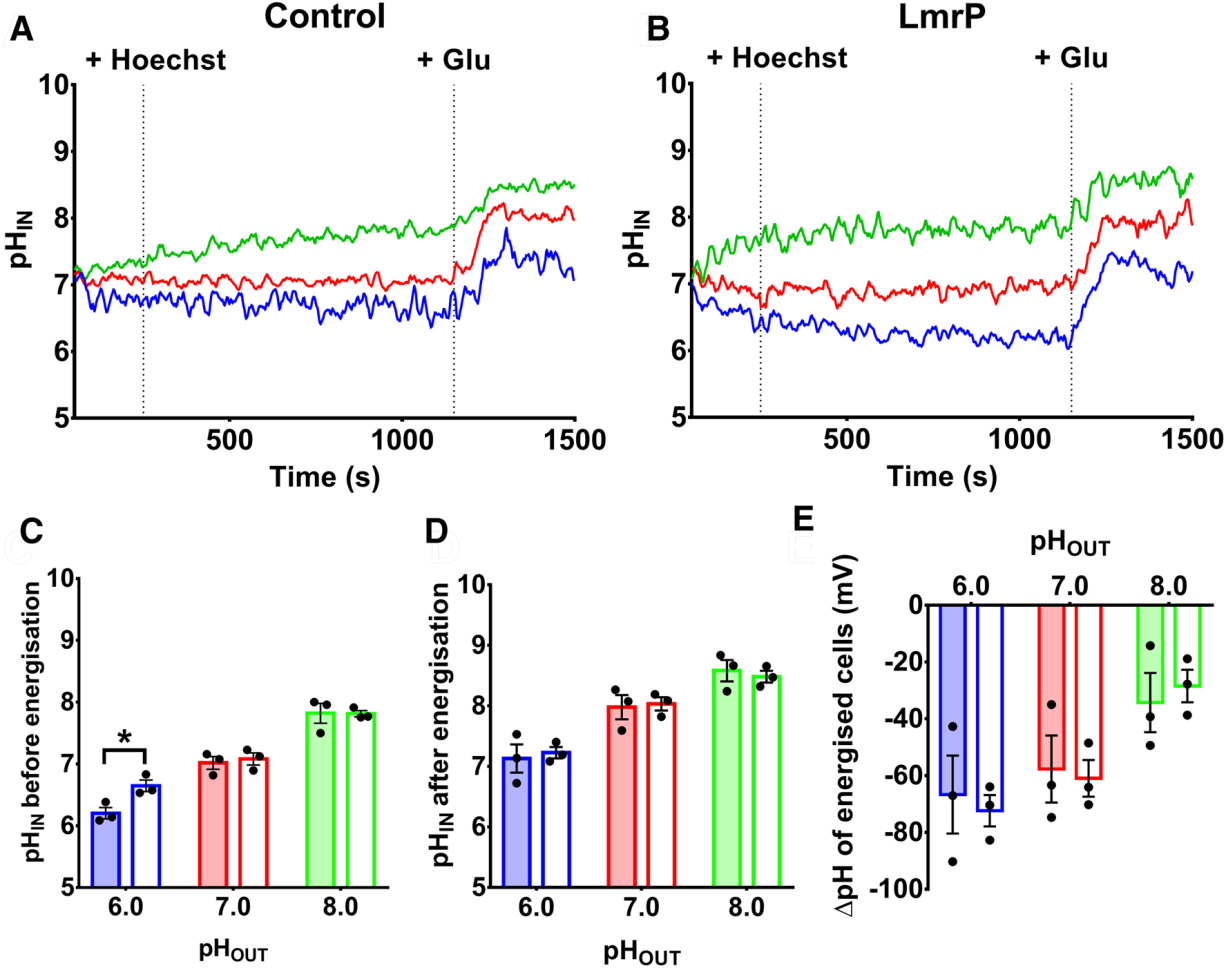
Intracellular pH changes in *L. lactis*. De-energised cells were loaded with 1 µM carboxyfluorescein succinimidyl ester (CFSE) and subsequently incubated with 25 mM glucose to allow the cells to generate metabolic energy. The 490:440 nm fluorescence ratio was followed over time and calibrated using the nigericin + high K^+^ method. **(A,B)** pH_IN_ measurements in control cells (panel **A**) and LmrP-expressing cells (panel **B**) at pH 6.0 (blue), 7.0 (red) and 8.0 (green). **(C,D)** pH_IN_ in LmrP-expressing and control cells (closed and open bars, respectively) before (panel **C**) and after (panel **D**) the addition of glucose. **(E)** ΔpH values calculated from the set pH_OUT_ and the observed pH_IN_ values (from panel **D**) using the simplified Nernst equation: $\Delta pH= 59\bullet ({pH}_{IN}-{pH}_{OUT})$. Bars in panels **(C–E)** represent mean ± S.E.M. of n = 3 independent experiments (two-way analysis of variance; * p < 0.05).

**Figure 5:**
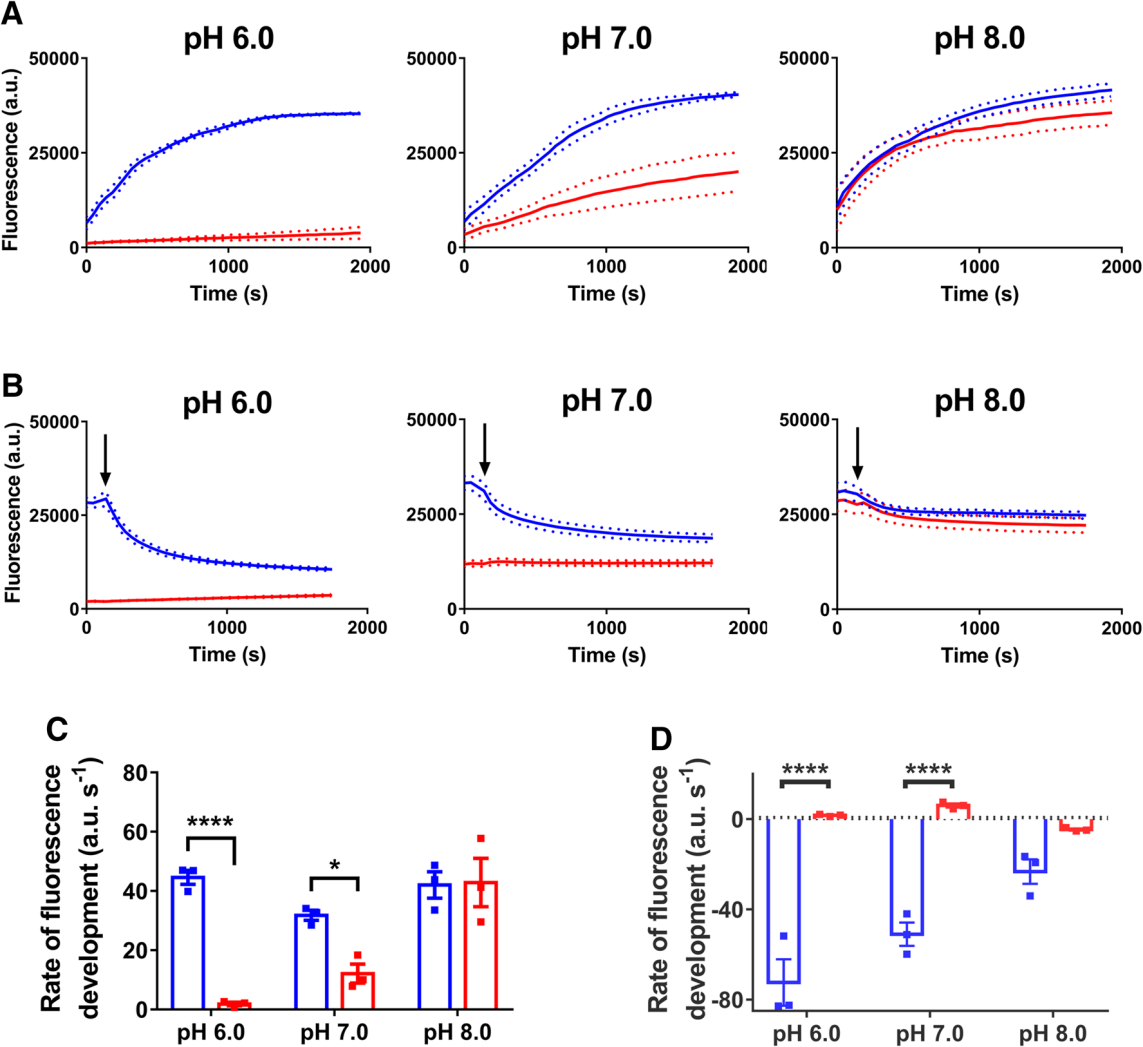
Uptake and efflux of Hoechst in cells. **(A)** Hoechst fluorescence in de-energised LmrP-expressing cells (blue) and control cells (red) at pH 6.0, 7.0 or 8.0 following the addition of 1 µM Hoechst (at start of time course). **(B)** Following the equilibration of Hoechst fluorescence, 25 mM glucose was added (at arrow) to allow the generation of metabolic energy in the cells, after which fluorescence changes were followed over time. **(C,D)** Rates of linear fluorescence increase over t = 0–322 s in panel A and linear fluorescence decrease over t = 138–184 s (corresponding to 46–92 s post glucose addition) in panel B. Solid and dotted lines in panel A represent mean ± S.E.M. of n = 3 independent experiments. Rate data are mean ± S.E.M from 3 independent experiments (two-way analysis of variance; * p < 0.05; **** p < 0.001).

Hoechst transport measurements in our lactococcal cells usually involve the uptake and build-up of an intracellular pool of Hoechst in cells that are low in metabolic energy, followed by the multidrug transporter-mediated efflux and emptying of this pool when cellular metabolism has been re-initiated by the addition of glucose. What happens in this protocol with Hoechst fluorescence in relation to changes in the intracellular and extracellular pH? To answer this question, we used cells containing a covalently-bound pH indicator, carboxyfluorescein diacetate succinimidyl ester (CFSE), in the intracellular environment, whose fluorescence emission (Fig. 4) can be measured independently from that of Hoechst (Fig. 5). For this purpose, *L. lactis* cells were incubated with CFSE after induction with nisin and then resuspended in fresh media to allow any free CFSE to be effluxed. Cells were washed once in KPi buffer (pH 7.0) and incubated with buffer containing 2,4-dinitrophenol (DNP), a protonophore that dissipates ΔpH. The intracellular ATP pool is subsequently depleted by compensatory H^+^ efflux by the F_0_F_1_ H^+^-ATPase^35^. The de-energised cells were then washed and diluted in external buffer with a fixed pH_OUT_ of 6.0, 7.0 or 8.0 to which 1 µM Hoechst was added. The pH-dependent fluorescence of CFSE during the Hoechst influx phase of the experiment indicated that the intracellular pH of de-energised cells generally approaches that of the external buffer over time due to the inability of the cells to maintain pH homeostasis in the intracellular environment (Fig. 4A,B). At pH_OUT_ 6.0, this process occurred more slowly for de-energised control cells than it did for cells expressing LmrP, resulting in a slightly higher pH_IN_ at the time point where glucose was added (Fig. 4C). Following the preloading of cells with Hoechst, the addition of glucose initiated a rapid alkalinisation of the cytoplasm relative to the external buffer (Fig. 4A-D) and generation of a larger ΔpH (interior alkaline) at pH_OUT_ 6.0 than at pH_OUT_ 8.0 (Fig. 4E). When taken together, the experiments in Fig. 4 show that at a fixed pH_OUT_, the pH_IN_ develops similarly over time in cells with or without the expression of LmrP.

In similar experiments, we also measured how the Hoechst fluorescence changes over time (Fig. 5). In the Hoechst uptake phase, LmrP-expressing cells exhibited a fast fluorescence development over time that was relatively independent of pH_OUT_ (Fig. 5A,C). In contrast, the rate of fluorescence development in control cells was very low at pH_OUT_ 6.0 and increased with increasing pH (Fig. 5A). Thus, the passive influx of Hoechst across the membrane is much more pH-sensitive than facilitated uptake by LmrP. With the higher abundance of lipid-intercalated Hoechst at pH_OUT_ 8.0 than 6.0 (Fig. 3), these data support a model where the passive influx of Hoechst across the plasma membrane is based on the flipping of intercalated Hoechst from the outer leaflet to the inner leaflet. In the facilitated influx reaction catalysed by LmrP, Hoechst is maintained in a transport-competent state through binding to the substrate-binding pocket in this enzyme.

After the fluorescence of cells preloaded with Hoechst reached a steady-state level, the addition of glucose allowed the cells to generate metabolic energy and initiate the Hoechst efflux phase in the experiment. LmrP-expressing cells exhibited a significant decrease in Hoechst fluorescence, the rate of which was largest at pH_OUT_ of 6.0 but close to zero at pH_OUT_ of 8.0 (Fig. 5B,D). In contrast, the rate in control cells was close to zero at all pH_OUT_ values (Fig. 5B,D). Given that LmrP is a proton/drug antiporter^24^, the highest efflux rates are achieved at pH_OUT_ 6.0, where the magnitude of the ΔpH is most substantial relative to pH_OUT_ 8.0 (Fig. 4E).

**Figure 6:**
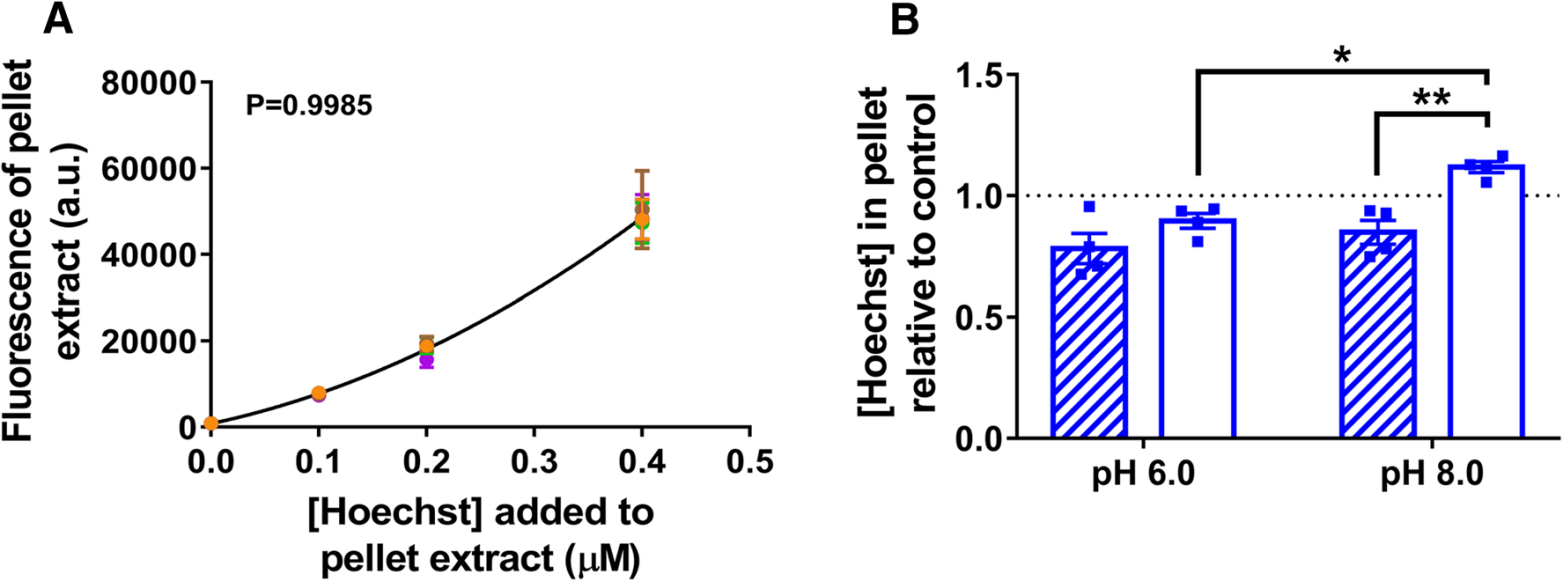
Quantitation of Hoechst in cells. Cells were spun down at 900 s after the addition of Hoechst (before glucose) in Fig. 5A, and 900 s after the addition of glucose in Fig. 5B. Hoechst was extracted from the cell pellets with isopropanol. **(A)** To produce calibration curves, identical cell suspensions without the addition of Hoechst underwent isopropanol extraction; before glucose, pH 6.0 (orange) or pH 8.0 (brown); after glucose, pH 6.0 (green) and pH 8.0 (purple). Subsequently, known concentrations of Hoechst were added to the pellet extracts, after which the fluorescence was recorded. The data points show overlap. A second-order polynomial curve was fitted to the data and used to interpolate the unknown Hoechst concentrations from the fluorescence in the test samples. The p-value quoted is the result of an extra sum-of-squares *F*-test to assess whether the differences between calibration curves were statistically significant. The threshold of significance was not reached, and thus a single curve was deemed adequate to interpolate all test fluorescence data. Data are presented as mean ± S.E.M from n ≥ 4 independent experiments. **(B)** The interpolated Hoechst concentration in pellet extracts of LmrP-expressing cells at pH 6.0 and pH 8.0, before (hatched bars) and after glucose (open bars) is shown relative to the amount in the extracts from control cells. Bars represent mean ± S.E.M from 4 independent experiments (two-way analysis of variance; * p < 0.05; ** p < 0.01). The unnormalised Hoechst quantitation data are presented in Supplementary Figure S1.

To assess how the differences in Hoechst fluorescence in cells correlate to differences in the actual amount of Hoechst in the cells, we extracted Hoechst from the cell pellet using isopropanol. These extractions were carried out 900 s after the start of the uptake phase in Fig. 5A and efflux phase in Fig. 5B. We then measured the Hoechst fluorescence emission in this hydrophobic solvent under standardised conditions using calibration curves that were highly reproducible (Fig. 6A). Surprisingly, there was no significant change in the amount of Hoechst associated with LmrP-expressing cells at pH 6.0 before or after glucose addition relative to control cells (Fig. 6B). Given the results of the Hoechst fluorescence measurements (Fig. 5B,D), these data indicate that during efflux at pH_OUT_ 6.0, LmrP converts a pool of fluorescent, DNA-bound and intercalated Hoechst in the inner membrane leaflet to a pool of non-fluorescent, membrane-bound Hoechst on the external membrane surface. Following the addition of glucose at pH 8.0, a significant increase in the amount of cell-associated Hoechst was observed for LmrP-expressing cells, suggesting that LmrP facilitates Hoechst uptake at pH 8.0 (Fig. 6B). As the Hoechst fluorescence measurements in cells at pH 8.0 did not reveal significant LmrP-mediated changes in fluorescence (Fig. 5), the measurements of actual Hoechst accumulation in cells provide more accurate information about the Hoechst transport reaction.

**Figure 7:**
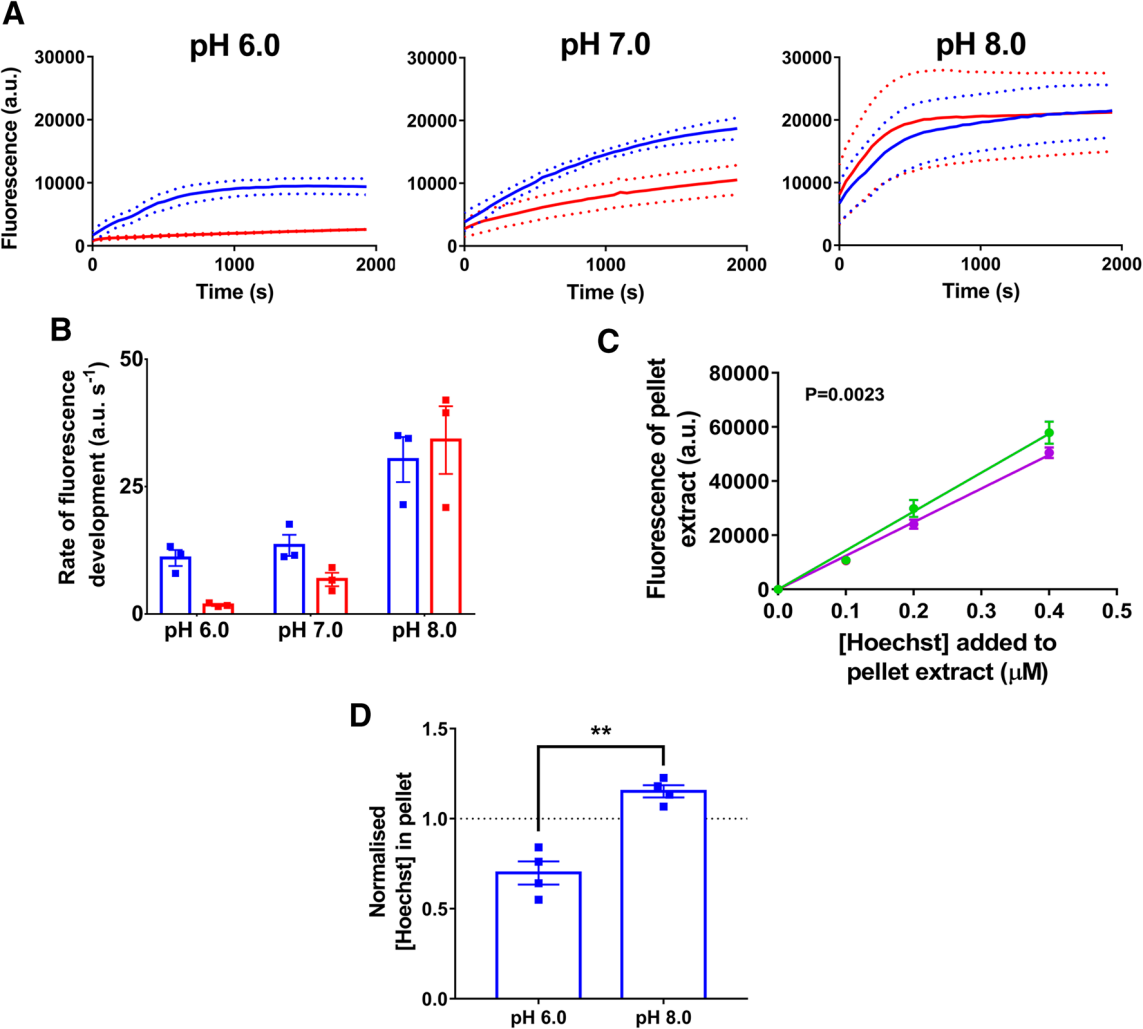
Accumulation-based Hoechst transport assay. **(A)** LmrP-expressing cells (blue) and control cells (red) were incubated in glucose-containing buffer at pH 6.0, 7.0 or 8.0. Following the addition of 1 µM Hoechst the fluorescence was followed over time. **(B)** Rate of linear fluorescence increase over t = 0–322 s in panel **(A)** for the two cell types at each pH. Colours are the same as those in panel **(A)**. In panel **(A)**, dotted lines represent mean ± S.E.M. of n = 3 independent experiments; in panel **(B)**, bars represent mean ± S.E.M. in 3 independent experiments. **(C,D)** Hoechst was extracted from the pellet of the cell suspensions, 900 s after the initial addition of Hoechst. **(C)** Calibration curves were produced using the same procedure as in Fig. 6, at pH 6.0 (green) or pH 8.0 (purple). Data are presented as mean ± S.E.M from n ≥ 6 independent experiments. **(D)** The interpolated amount of Hoechst in the pellet of LmrP-expressing cells is shown relative to the amount in the extracts from control cells. Bars are the mean ± S.E.M from 4 independent experiments (Welch’s *t*-test in panels E and F; ** p < 0.01). The unnormalised Hoechst quantitation data are presented in Supplementary Figure S2.

We also measured Hoechst fluorescence and quantified the amount of Hoechst in a frequently used drug accumulation assay in glucose-energised cells. In this type of assay, drug efflux by the expressed multidrug transporter is indirectly derived from the reduced drug accumulation compared to the non-expressing control. Here, the direct measurements of the amount of Hoechst in cell pellets revealed elevated levels in energised LmrP-expressing cells at pH_OUT_ 8.0 relative to pH_OUT_ 6.0 (Fig. 7D). Consistent with the data for glucose-energised LmrP-expressing cells in Fig. 6, these data suggest that the ΔpH (interior alkaline)-dependent extrusion by LmrP at pH 6.0 is reversed at pH 8.0, causing LmrP-mediated Hoechst uptake. However, in the Hoechst fluorescence measurements in this assay, LmrP expression was associated with *elevated* Hoechst fluorescence relative to control at pH 6.0, and an unaltered fluorescence compared to control at pH 8.0 (Fig. 7A,B). When taken together, these experiments show that the changes in the intensity of Hoechst fluorescence in cells over time do not always correlate well with changes in the amount of Hoechst associated with these cells.

## Discussion

**Figure 8:**
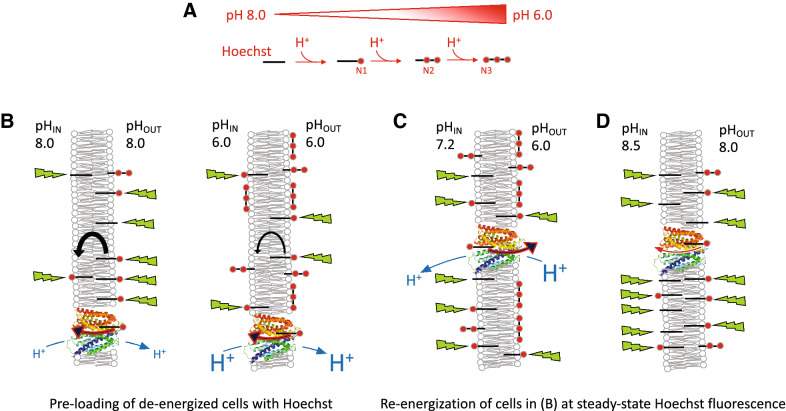
Schematic explaining the effects of pH on Hoechst transport across the plasma membrane. **(A)** Hoechst (*short black lines*) contains three basic nitrogen atoms with pKa values of 7.87 (N_1_), 5.81 (N_2_) and 5.02 (N_3_). Protonation of N_1_–N_3_ is indicated with *red circles*. Unprotonated and N_1_-protonated Hoechst can intercalate in the membrane and emit a high level of fluorescence in this hydrophobic environment. Conversely, Hoechst protonated on the N_1_ + N_2_ and N_1_ + N_2_ + N_3_ positions do not intercalate in a similar fashion but bind to the membrane surface where they remain exposed to the solvent and emit fluorescence at a low level. **(B)** Passive and LmrP-mediated (facilitated) influx of Hoechst into cells. As de-energised cells are unable to maintain ΔpH, the pH_IN_ approximately equals pH_OUT_. During passive Hoechst uptake at pH_OUT_ 8.0, a large majority of Hoechst molecules intercalates in the outer membrane leaflet from where the dye flips to the inner membrane leaflet. As the fraction of intercalated Hoechst is only small at pH_OUT_ 6.0, passive uptake is relatively slow. However, as LmrP selectively binds intercalated Hoechst from the membrane, the facilitated uptake of Hoechst by drug/H^+^ antiport is less dependent on substrate availability, and hence, on pH_OUT_. **(C)** Active efflux of Hoechst by LmrP from cells that are preloaded with Hoechst. When metabolising glucose, cells establish a ΔpH (interior alkaline). At pH_OUT_ 6.0/pH_IN_ 7.2, which corresponds to a significant inwardly-directed chemical proton gradient of −71 mV, the fluorescence measurements in cells indicate that LmrP mediates Hoechst/proton antiport in a ΔpH (interior alkaline)-dependent fashion. In this reaction, highly-fluorescent intercalated Hoechst is transported from the inner membrane leaflet to the relatively acidic external environment, where lowly-fluorescent multiprotonated Hoechst species adhere to the external membrane surface. **(D)** At pH_OUT_ 8.0/pH_IN_ 8.5, where the proton concentrations and ΔpH are low, but the inwardly-directed Δψ (interior negative) is large, an increase in cell-associated Hoechst is observed for LmrP-expressing cells.

In our measurements of Hoechst binding to DNA, the fluorescence remained relatively constant above pH 7.0 (Fig. 2). Therefore, Hoechst binding to DNA in many prokaryotic and eukaryotic cells is unlikely to be affected significantly by pH_IN_ within their typical physiological range. The fluorescence of Hoechst added to our liposome suspensions showed an increase as buffer pH increased from pH 6.0 up to 7.5 (Fig. 3A). The inclusion of DNA in the lumen of liposomes did not significantly change the pH profile of Hoechst fluorescence, pointing to the dominance of Hoechst-membrane interactions to the fluorescence emission in this system. This is in contrast to ethidium, which is also used for transport assays in DNA-loaded proteoliposomes^25^. For ethidium, the majority of fluorescence comes from DNA binding, as the fluorescence emission of lipid-associated dye is low. The increase in Hoechst fluorescence in liposome suspensions with buffer pH from 6.0 to 7.5 (Fig. 3A) is consistent with the dominance of di- or trivalent cationic Hoechst species at low pH (Fig. 1), which are unable to insert between phospholipids due to the presence of positive charges along the entire drug molecule (see schematic in Fig. 8A). Our supernatant data in liposomes (Fig. 3B) show that these cationic species adhere well to the membrane surface, which is most likely based on electrostatic interactions with the negatively charged phosphate moieties near the phospholipid headgroups. As pH increases, Hoechst deprotonation is favoured. Monovalent cationic Hoechst with protonation at N_1_ (Fig. 1) and unprotonated Hoechst can now intercalate effectively between the phospholipids, which is associated with a dramatic increase in fluorescence per molecule of liposome-associated Hoechst.

The pH dependence of Hoechst fluorescence in liposomes is also observed in control cells (Figs. 5 and 7). Hoechst fluorescence was found to increase with increasing pH_OUT_ of the cell suspension from 6.0 to 8.0. As energised cells, with or without LmrP expression, generally have similar pH_IN_ values at varying pH_OUT_ (Fig. 4D), changes in Hoechst fluorescence over time between the two cell types will be LmrP-dependent. At pH 8.0, LmrP expression had little effect on the fluorescence of de-energised cells exposed to Hoechst (Fig. 5A). This is consistent with the notion that high pH favours the formation of Hoechst species that intercalate well within the membrane and are highly fluorescent in this environment (Fig. 8B). In contrast, de-energised control cells exhibited negligible fluorescence at pH 6.0 (Fig. 5A), where a majority of Hoechst species is predicted to be di- or trivalent cationic (Fig. 1B) and effectively binds to the membrane surface rather than intercalating in the phospholipid bilayer. Conversely, de-energised LmrP-containing cells at pH_OUT_ 6.0 rapidly developed high Hoechst fluorescence (Fig. 5A). We propose that LmrP-mediated, facilitated Hoechst influx into the cell (Fig. 8B) leads to a net insertion of the dye into the inner membrane leaflet, an environment where Hoechst is highly fluorescent. This is the reverse of the reaction scheme for LmrP-mediated efflux of the water-insoluble membrane-fluidity-probe TMA-DPH, in which this probe is actively transported from the inner leaflet of the membrane to the outer leaflet^36^. Subsequently, the pool of Hoechst in the inner leaflet of the membrane will partly diffuse into the cytosol and equilibrate with a pool of Hoechst bound to chromosomal DNA.

The addition of glucose to de-energised lactococcal cells rapidly allows the synthesis of ATP and re-establishment of a PMF through proton pumping by the F_0_F_1_ H^+^-ATPase in the plasma membrane. At pH_OUT_ 6.0, cells generate the largest inwardly-directed ΔpH (interior alkaline) (Fig. 4E), which promotes drug-proton antiport by LmrP and favours the LmrP-mediated efflux of intercalated Hoechst from the inner membrane leaflet (Fig. 5B). This reaction will also empty the pool of DNA-bound Hoechst due to Hoechst re-partitioning into the inner membrane leaflet. As LmrP mediates Hoechst efflux into the relatively-acidic extracellular environment, where cationic Hoechst species adhere to the external membrane surface (Fig. 3), there is little detectable change in the amount of cell-associated Hoechst during transport (Fig. 6B). This mechanism (Fig. 8C) is also relevant for observations on the fluorescence quenching associated with Hoechst transport from the external buffer into the acidic lumen in lactococcal inside-out membrane vesicles^37^, in which an outwardly-directed PMF (interior positive and acidic) is maintained due to proton pumping by the F_0_F_1_ H^+^-ATPase^38^. As we do not see significant LmrP-mediated efflux of Hoechst in cells at pH 8.0 (Fig. 6B), where the Δψ is the major contributor to the PMF, the Δψ does not appear to be a driving force for LmrP-mediated Hoechst efflux at this pH. It is, therefore, likely that Hoechst extrusion by LmrP generally proceeds via an electroneutral antiport mechanism. Given the flexibility in proton coupling in LmrP, with 2H^+^/ethidium but 3H^+^/propidium^24^, it is not yet possible to say which cationic Hoechst species are substrates of LmrP. However, the observations of Hoechst transport at pH 7.0, where nearly all Hoechst is monovalent cationic, make this species a likely candidate.

In the Hoechst accumulation assays (Fig. 7), energised LmrP-expressing cells at pH_OUT_ 6.0 developed a higher level of Hoechst fluorescence than control cells (Fig. 7A). However, measurements of the amount of cell-associated Hoechst at this pH show that the LmrP-expressing cells did not accumulate more Hoechst than control cells. On the contrary, the accumulation level tended to be lower due to ΔpH (interior alkaline)-dependent Hoechst extrusion by LmrP (Fig. 7D). Therefore, the Hoechst molecules in these LmrP-expressing cells are more fluorescent than those associated with control cells. As the pH_IN_ values of both types of energised cells are essentially similar at the same pH_OUT_ (Fig. 4D), we propose that, by catalysing Hoechst binding in its interior cavity and dissociation towards the membrane, outward-facing LmrP rapidly re-orients surface-bound Hoechst in the outer membrane leaflet to intercalated Hoechst in this leaflet. At a pH_OUT_ of 6.0, this effect might also contribute to the increased Hoechst fluorescence in de-energised LmrP-containing cells relative to the control cells (Fig. 5A).

Consistent with a previous study^26^, the amount of Hoechst associated with LmrP-expressing cells increased significantly at pH_OUT_ 8.0 when cells generated metabolic energy from glucose catabolism (Figs. 6B and 7D). This increase was not associated with a significant enhancement in the Hoechst fluorescence in the cells (Figs. 5B and 7A) due to the very high pH_IN_ of 8.5 at pH_OUT_ of 8.0 (Fig. 4D) and the significant decline in the fluorescence of membrane-bound Hoechst at pH values above 8.0 (Fig. 3A). This means that, although the amount of Hoechst in LmrP-expressing cells at pH_OUT_ 8.0 is higher than control cells (Figs. 6B and 7D), any intracellular Hoechst will have a local pH of ~ pH 8.5 (Fig. 4D) and thus exhibit lower fluorescence than extracellular Hoechst with local pH of pH 8.0 (Fig. 3A). Therefore, control cells show similar fluorescence to LmrP-expressing cells, despite having taken up less Hoechst (Figs. 5B and 7A). Furthermore, the alkaline pH_OUT_ and pH_IN_ facilitate intercalation of Hoechst in the membrane and passive flipping of the dye to the cellular interior, which reduces the contribution of LmrP activity to the fluorescence changes (Fig. 8D). The mechanism of LmrP-dependent Hoechst uptake might relate to published findings^27,28^ that Hoechst binding to purified LmrP at pH 8.0 stabilises an outward-facing conformation rather than the inward-facing conformation. Together with our results, this suggests that Hoechst initially binds to LmrP at the outside of the membrane from where transport is initiated, leading to LmrP-mediated Hoechst uptake. This aspect of LmrP-mediated Hoechst transport requires further study.

In conclusion, we have analysed the effects of pH on Hoechst 33342 transport by the MFS transporter LmrP in Gram-positive *L. lactis*. We demonstrate that changes in Hoechst fluorescence in cells can be used to assess multidrug transport activity but that quantitative conclusions about the direction and rate of this activity cannot rely on fluorescence measurements alone. To complement such analyses, we established a novel isopropanol extraction method to determine the amount of Hoechst in cells directly. Using both approaches, we describe the ΔpH (interior alkaline)-dependent Hoechst efflux by LmrP as a reaction in which a highly-fluorescent pool of membrane-intercalated Hoechst in the cellular interior is converted into a lowly-fluorescent pool of membrane-surface-bound Hoechst at the extracellular side of the plasma membrane. Furthermore, we observe LmrP-mediated Hoechst uptake in cells under conditions where the ΔpH is very small and the Δψ (interior negative) is substantial. Our methods and conclusions are directly relevant for the transport of amphiphilic antibiotics, antineoplastic agents and cytotoxic compounds that are differentially protonated within the physiological pH range.

## Methods

### Materials

All compounds and chemicals were purchased from Sigma-Aldrich unless indicated otherwise.

### Hoechst speciation

Calculations of pKa values of Hoechst and species distribution as a function of pH were performed using Chemicalize, Jan 2020, https://chemicalize.com/ developed by ChemAxon (https://www.chemaxon.com).

### Hoechst fluorescence in buffer with and without DNA

One µM Hoechst 33342 or 2 µM ethidium bromide was added to Assay Buffer (20 mM (K)MES-PIPES-HEPES adjusted to pH 6.0 to 9.0 by the addition of aliquots of 5 M KOH) in the wells of a 96-well plate with and without 1 mg mL^−1^ sheared calf thymus DNA (ThermoFisher). The fluorescence was measured in a Clariostar Plus plate reader (BMG Labtech). Hoechst fluorescence was measured with excitation and emission wavelengths of 355 nm and 460 nm, respectively. Ethidium bromide fluorescence was measured in 96-well black-walled and bottomed plates with excitation and emission wavelengths of 500 and 580, respectively. All excitation and emission used slit widths of 20 nm.

### Hoechst transport in cells

*Lactococcus lactis* NZ9000 Δ*lmrA* Δ*lmrCD* harbouring empty control vector (pNZ8048), or pHLP5 containing the *lmrP* gene under the control of a nisin-inducible promoter, were grown overnight at 30 °C in complete M17 medium supplemented with 25 mM glucose and 5 µg mL^−1^ chloramphenicol. These cultures were used to inoculate 50 mL of pre-warmed complete M17 medium. Cells were grown to an OD_660_ of 0.55, after which protein expression was induced for 2 h by the addition (1:1000 v/v) of the culture supernatant from the nisin A-producing *L. lactis* strain NZ9700 which was grown for 2 h at 30 °C in M17 medium supplemented with 25 mM glucose only. LmrP-expressing cells or control cells were harvested by centrifugation (6500 × *g*, 10 min) and washed once with ice-cold 50 mM KPi buffer supplemented with 5 mM MgSO_4_ at pH 7.0. Cells were then incubated in KPi buffer of the same composition with the addition of 0.5 mM 2,4-dinitrophenol (40 min, 30 °C) to deplete intracellular ATP concentrations. Following this, cells were centrifuged (6500 × *g*, 10 min) and then washed three times with ice-cold KPi buffer at pH 7.0. After the final wash, cells were resuspended in KPi buffer to an OD_660_ of 5 and kept on ice for a maximum of 2 h. Cells were then diluted tenfold in KPi buffer of varying pH values in a 96 well plate. For de-energised accumulation, 1 µM Hoechst was added after which dye fluorescence was followed over time in a plate reader using equipment and settings as described under “Hoechst fluorescence in buffer with and without DNA”. When steady-state fluorescence was reached, 25 mM glucose was added to enable the generation of metabolic energy in the cells. For energised accumulation of Hoechst in cells, 25 mM glucose was added first, and the cells were incubated for 3 min before the addition of 1 µM Hoechst. The rate of fluorescence development during accumulation and efflux was calculated by calculating the gradient of the linear portion of the fluorescence trace over a time span as indicated in the legend to Figs. 5 and 7.

### Preparation of liposomes and proteoliposomes containing purified LmrP

After nisin-induced protein expression in *L. lactis*, cells were treated with lysozyme and then disrupted using a Basic Z 0.75 kW Benchtop Cell Disruptor (Constant Systems) as described previously^24^. The resulting inside-out membrane vesicles were solubilised at a protein concentration of 12 mg mL^−1^ in 50 mM KPi buffer (pH 8.0) containing SIGMAFAST EDTA-free protease inhibitor cocktail, 100 mM NaCl, 10% v/v glycerol and 1% β-d-dodecyl maltoside (DDM) (Anatrace). His_6_-tagged LmrP was purified using Ni^2+^-NTA affinity chromatography^25^. Liposomes and proteoliposomes were prepared as described^39^. Briefly, a 3:1 mixture of acetone-diethylether washed total *E. coli* lipids and egg-yolk phosphatidylcholine in chloroform (w/w, total 4 mg mL^−1^, Avanti Polar Lipids Inc.) was dried using N_2_ gas and rehydrated with Assay Buffer at pH 7.0 (see under “Hoechst fluorescence in buffer with and without DNA”). The lipid was extruded 11 times using a 400 nm polycarbonate filter. For liposomes containing DNA, 1 mg mL^−1^ sheared calf thymus DNA was added to the internal buffer before extrusion. Subsequently, the liposomes were incubated with 10 mM MgSO_4_ plus 10 mg mL^−1^ DNase to remove DNA contamination at the external membrane surface. For reconstitution of LmrP, Triton X-100-destabilised liposomes were mixed with affinity-purified protein at a lipid: protein ratio of 50:1 (w/w). The detergent was removed using Bio-Beads SM-2 (Bio-Rad) after which the proteoliposomes were harvested by centrifugation (130,000 × *g*, 40 min, 4 °C). Liposomes and proteoliposomes were resuspended in Assay Buffer to OD_540_ of 0.5.

### Direct quantitation of Hoechst in cells and (proteo)liposomes

Lactococcal cells at OD_660_ of 5.0 were diluted 10 times in 1 mL KPi buffer at varying pH in 2 mL plastic tubes. For quantitation of energised accumulation, cells were incubated with 25 mM glucose for 3 min, and then test samples were allowed to equilibrate with 1 µM Hoechst for 15 min (shaking at 1000 rpm, 30 °C). The suspensions were transferred to clean tubes and centrifuged (5500 × *g*, 5 min). The supernatant was thoroughly removed from the cell pellet. The pellet was resuspended in a small volume of isopropanol (150 µL), before being transferred to a new tube and topped up to a volume of 350 µL. Tubes were then vortexed for 5 min to extract cell-associated Hoechst, before centrifugation (14,000 × *g*, 5 min) to pellet debris. 250 µL of the resulting supernatant was transferred to a 96-well plate. Isopropanol was added 1:1 (v/v), and the fluorescence measured in a plate reader. In parallel, cell suspensions identical to these test samples were prepared, minus the addition of Hoechst. The cell pellet from these samples was also collected, added to the same plate as the test samples and mixed with isopropanol. These wells were supplemented with known concentrations of Hoechst to allow the generation of calibration curves, which were prepared for each plate and all relevant combinations of pH and energisation. An identical protocol was followed to extract Hoechst from the pellet of de-energised cells (which were incubated with Hoechst for 15 min before centrifugation), and for cells subsequently re-energised with glucose (which were harvested 15 min after glucose addition).

For measurements of Hoechst fluorescence in (proteo)liposomes, these were prepared as described under “Preparation of liposomes and proteoliposomes containing purified LmrP” and diluted 100-fold in 2 mL of Assay Buffer pH 5.75 containing 1 µM nigericin and 0.1 µM Hoechst. The pH of the buffer was changed by the stepwise addition of 5 M KOH, after which the fluorescence was allowed to stabilise. Hoechst fluorescence was measured every second in an LS-55B luminescence spectrometer (Perkin-Elmer Life Sciences) with excitation and emission wavelengths of 355 nm and 460 nm respectively, and with slit widths of 10 nm and 4 nm, respectively. The steady-state fluorescence after each pH step was recorded. To measure Hoechst concentrations in the external buffer of liposome suspensions, empty liposomes were diluted 20-fold in 2 mL of Assay Buffer containing 0.1 µM Hoechst and 1 µM nigericin at each pH step. Following a 10 min incubation, the samples were centrifuged (130,000 × *g*, 40 min, 4 °C), and the supernatant was collected and transferred to wells of a 96-well plate to which the isopropanol was added. For calibration curves, identical supernatant samples were prepared in parallel from liposome suspensions to which no Hoechst had been added. Known concentrations of Hoechst were then added to produce calibration curves. Hoechst fluorescence was measured using equipment and settings as described under “Hoechst fluorescence in buffer with and without DNA”.

### Measurements of intracellular pH in cells

After membrane-permeable carboxyfluorescein diacetate succinimidyl ester (CFDA-SE, ThermoFisher) enters the cytoplasm of cells, intracellular esterases remove the acetate groups and liberate the CFSE probe. The latter covalently binds to intracellular macromolecules and displays pH-dependent fluorescence. The probe was used to monitor intracellular pH over the course of accumulation of 1 µM Hoechst followed by active Hoechst efflux in the presence of 25 mM glucose. For this purpose, the protocol in “Direct quantitation of Hoechst in cells and (proteo)liposomes” was modified in accordance with a previously described method^40^. Cell cultures received 1 µM CFDA-SE after 90 min of nisin-based induction of LmrP-expression in *L. lactis* and were further incubated for the remaining 30 min (30 °C). Cells were harvested by centrifugation (6500 × *g*, 10 min), and resuspended in fresh, pre-warmed M17 media containing 25 mM glucose and 5 mg mL^−1^ chloramphenicol (30 °C, 30 min), to allow the potential efflux of unbound CFSE by multidrug transporters. Cells were then de-energised, washed and resuspended to an OD_660_ of 5.0 for use in fluorescence assays. Samples were excited at both 490 nm and 440 nm, each with 5 nm slit width, in an LS-55B fluorimeter (see under “Hoechst transport in liposomes and proteoliposomes containing purified LmrP”). The emission was measured at 525 nm with 10 nm slit width. When CFSE is excited at 490 nm, the fluorescence emission varies with pH, while emission caused by excitation at 440 nm (the isosbestic point) is relatively insensitive to pH. Thus, the ratio of emission when excited at the two wavelengths (490:440) was used to correct for different CFSE loading levels in different cell batches. Conversion of fluorescence to pH was based on calibration curves that were prepared using the nigericin—high K^+^ method. In this method, LmrP-expressing cells and control cells were diluted in 50 mM KPi buffer at various pH values. Subsequently, 1 µM nigericin was added. As the external buffer contains high concentrations of K^+^, pH_IN_ rapidly equilibrates with pH_OUT_ due to electroneutral H^+^–K^+^ exchange via nigericin. The blanked CFSE fluorescence ratio was then used to calibrate pH_IN_ as a function of pH_OUT._ Transmembrane ΔpH was calculated in mV using the Nernst equation:

$$\Delta E= -\frac{RT}{zF}\bullet ln\frac{{[X]}_{OUT}}{{[X]}_{IN}}$$

where *R* is the universal gas constant, *T* is the absolute temperature in Kelvin, *z* is the valency of the ion in question, *F* is the Faraday constant and *[X]*_*out*_ and *[X]*_*in*_ are the extra- and intracellular concentrations of the ion respectively. For protons at 24 °C, the equation simplifies to:

$$\Delta E=-59\bullet ({\mathrm{log}}_{10}{\left[H\right]}_{OUT}-{\mathrm{log}}_{10}{\left[H\right]}_{IN})= 59\bullet \left({pH}_{OUT}-{pH}_{IN}\right)(\mathrm{mV})$$

### Statistical analyses

All numerical data are expressed as mean ± standard error of the mean (S.E.M.). All statistical tests were performed using GraphPad Prism 7.00, and p < 0.05 was considered statistically significant. Two-way ANOVA was used to assess the impact of experimental factors in experiments based on whole-cell fluorescence (those testing internal pH and fluorescence development upon Hoechst exposure). In these experiments, the significance of differing means was assessed post-hoc using Sidak’s multiple comparisons test. In isopropanol extraction experiments concerning de-energised accumulation and efflux, the same process was followed. In isopropanol extraction experiments concerning energised accumulation of Hoechst, normalised values for LmrP-expressing cells were compared using Welch’s *t*-test. In calibration curves used to quantify Hoechst in cell pellet samples, an Extra Sum-of-Squares F test was used to compare the regression models; if not significant, a single regression line was fitted to all data.

## Supplementary information

Supplementary Figures.

## Data Availability

Data that support the findings of this study have been deposited in the University of Cambridge research repository Apollo with DOI link https://doi.org/10.17863/CAM.58079 or are available from the corresponding author upon reasonable request.
