# Supplementary material for: Complexities of a protonatable substrate in measurements of Hoechst 33342 transport by multidrug transporter LmrP
Source: Sci Rep. 2020 Nov 18;10:20026. doi: 10.1038/s41598-020-76943-0 (PMC7674423; doi:10.1038/s41598-020-76943-0)
Supplement: Supplementary file 1 — Supplementary Figures. [file 41598_2020_76943_MOESM1_ESM.docx]

**SUPPLEMENTARY MATERIAL**

**Complexities of a protonatable substrate in measurements of Hoechst 33342 transport by multidrug transporter LmrP**

Brendan M. Swain^a^, Dawei Guo^a^, Himansha Singh^a^, Philip B. Rawlins^b^, Mark McAlister^b^ and Hendrik W. van Veen^a*^

*^a^Department of Pharmacology, University of Cambridge, Cambridge CB2 1PD, UK*

*^b^Structure, Biophysics & Fragment-Based Lead Generation, Discovery Sciences, BioPharmaceuticals R&D, AstraZeneca, Cambridge CB4 0WG, UK*


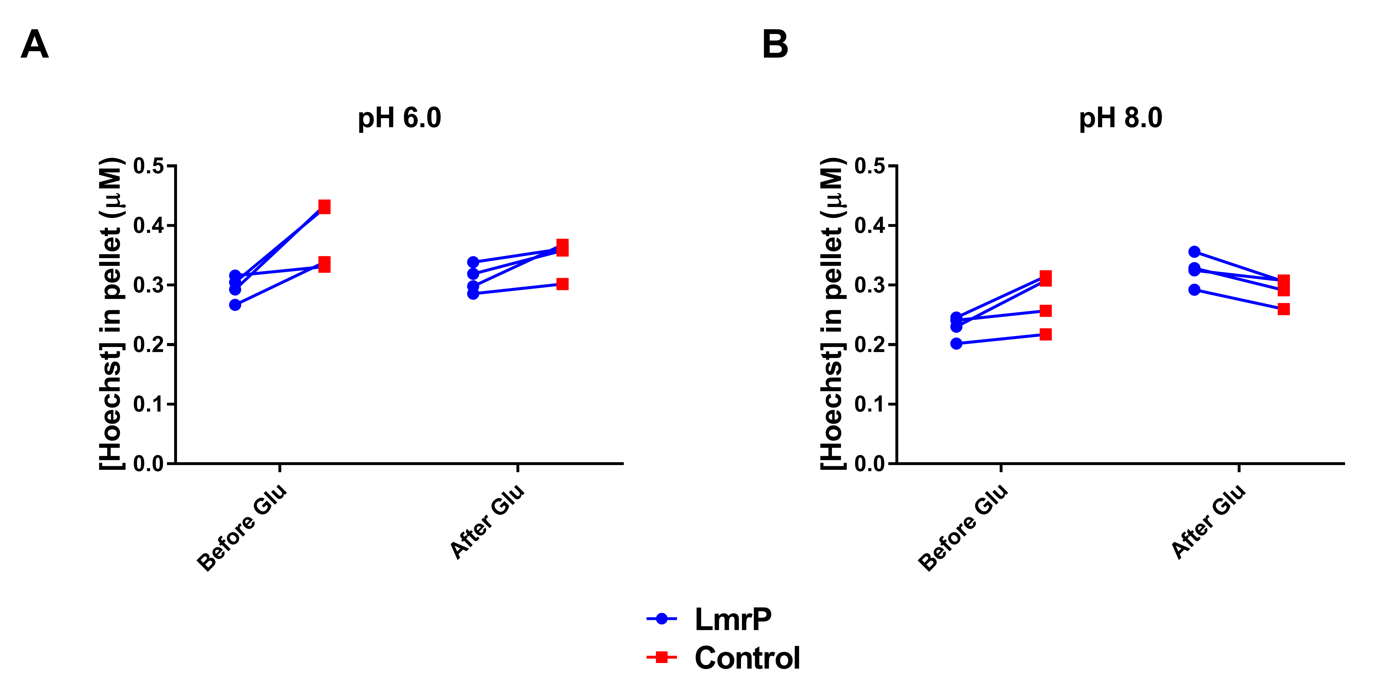


**Supplementary Figure S1.** Unnormalised Hoechst quantitation data for de-energised cells before and after glucose addition. The fluorescence intensity in Hoechst extracts prepared for Fig. 6B was interpolated to concentration (µM) using the corresponding calibration curves in Fig. 6A and plotted without further normalisation. The individual data points represent the Hoechst concentration in extracts from LmrP-expressing cells (blue) or control cells (red), 900s after the addition of 1 µM Hoechst (“before Glu”) and 900s after the addition of glucose (“after Glu”). Data are shown for cell pellets at pH 6.0 (panel A) and 8.0 (panel B). Samples from the same biological replicate are joined by a line.


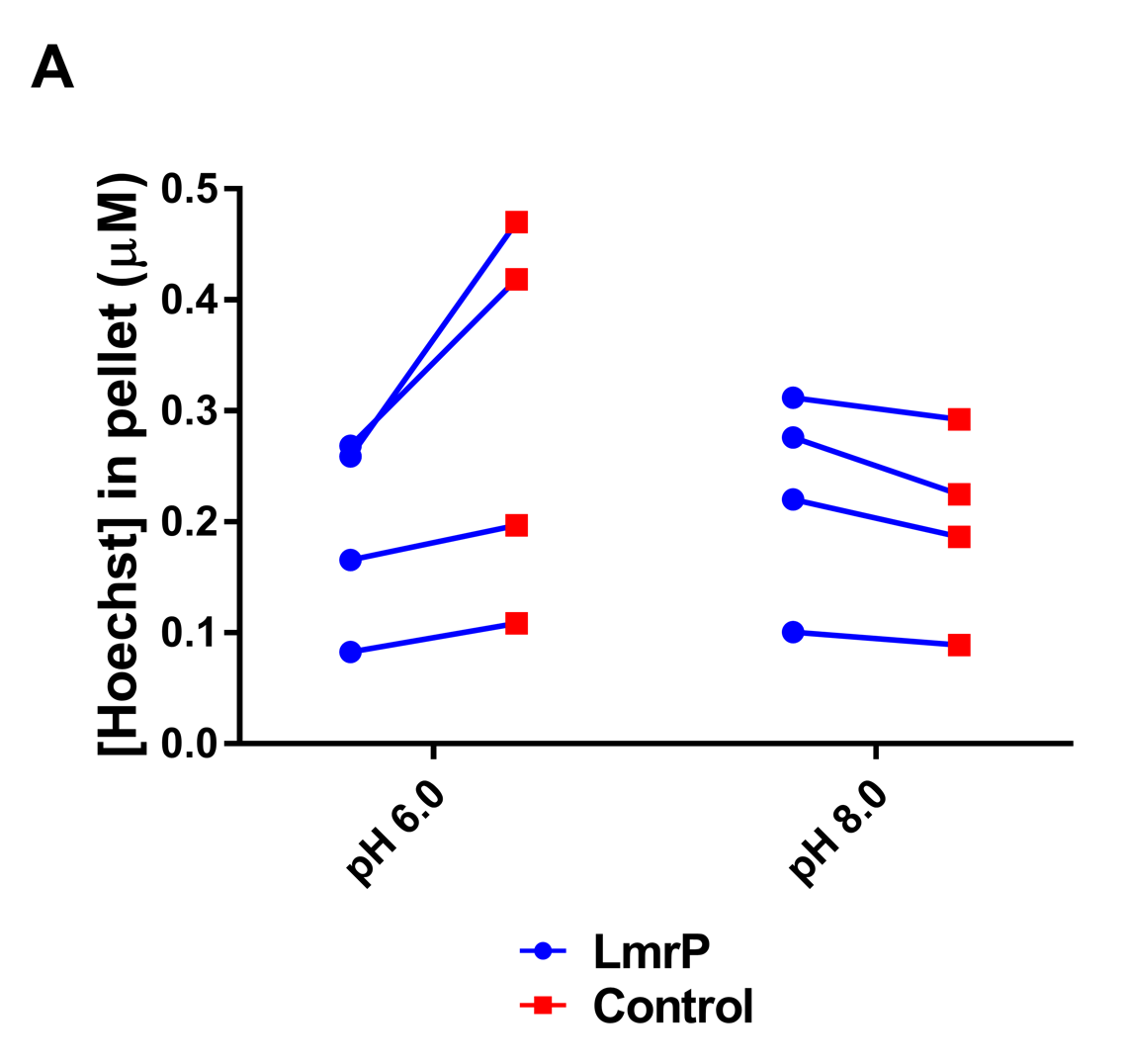


**Supplementary Figure S2.** Unnormalised Hoechst quantitation data for accumulation experiments with energised cells. The fluorescence intensity of the Hoechst extracts in Fig. 7D was interpolated to concentration (µM) using the corresponding calibration curves in Fig. 7C and plotted without further normalisation. The data points represent the Hoechst concentration in pellet extracts from energised cells, 900s after the addition of 1µM Hoechst at either pH 6.0 or pH 8.0. LmrP-expressing (blue) and control cells (red) from the same biological replicate are joined by a line.
